# The coupling of the M2 muscarinic receptor to its G protein is voltage dependent

**DOI:** 10.1371/journal.pone.0224367

**Published:** 2019-10-31

**Authors:** Yair Ben-Chaim, Chava Broide, Hanna Parnas

**Affiliations:** 1 Department of Natural and Life Sciences, The Open University of Israel, Ra'anana, Israel; 2 Department of Neurobiology, Institute of Life Sciences, The Hebrew University, Jerusalem, Israel; Memorial Sloan-Kettering Cancer Center, UNITED STATES

## Abstract

G protein coupled receptors (GPCRs) participate in the majority of signal transduction processes in the body. Specifically, the binding of an external agonist promotes coupling of the GPCR to its G protein and this, in turn, induces downstream signaling. Recently, it was shown that agonist binding to the M2 muscarinic receptor (M2R) and to other GPCRs is voltage dependent. Here we examine, whether the coupling of the M2R to its G protein is also voltage-dependent. We first show, in Xenopus oocytes, that the activity of the M2R in the absence of agonist (constitutive activity) can be used to report the coupling. We then show that the coupling is, by itself, voltage dependent. This novel finding is of physiological importance, as it shows that the actual signal transduction, whose first step is the coupling of the GPCR to its cognate G protein, is voltage dependent.

## Introduction

Binding of agonists to GPCRs promotes coupling of the latter to their G protein and this, in turn, induces downstream signaling [1]. The binding of agonists to GPCRs [2–7], and the efficacy of agonists [8–10], was shown to be voltage dependent We provided experimental evidence indicating that the coupling of the M2R to its G protein plays a role in controlling the voltage dependence of agonist binding to the M2R [2,3,11]. Based on these results we hypothesized that the coupling itself may be voltage dependent [11]. To test this hypothesis, it is necessary to examine the coupling itself.

The M2R, as well as other GPCRs, induces downstream signaling even in the absence of agonist. This activity, termed "constitutive activity", was interpreted to be a result of a spontaneous coupling of the receptor to its G protein [12–15]. Therefore, constitutive activity measurements may be used as a new experimental paradigm to examine the coupling itself.

To measure the constitutive activity we used, as in our earlier studies [3,11], Xenopus oocytes as an expression system and M2R-activated GIRK (G protein-activated inward rectifying K^+^ channel) currents as a measure for M2R signaling.

Here we show that the M2R exhibits constitutive activity. We further show that this constitutive activity reflects the coupling of the M2R to its cognate G protein. We finally show that the coupling is voltage dependent.

## Materials and methods

Frogs were used according to guidelines of the Hebrew University in Jerusalem and studies were approved by the Institutional Animal Care and Use Committee. *Xenopus* oocytes were prepared, injected, and maintained as previously described [16,17]. Constructs were linearized and transcribed as previously described [18]. The triple mutant was constructed using QuikChange site-directed mutagenesis kit (Agilent technologies, CA, USA).

### GIRK current measurements

Currents were recorded using the standard two-electrode voltage-clamp technique (Axoclamp 2B amplifier; Axon Instruments). pCLAMP8 software (Axon Instruments) was used for data acquisition and analysis. G protein-activated inwardly rectifying K^+^ (GIRK) currents were measured as described [3]. Briefly, oocytes were injected with 2 ng of the M2R (wt or the triple mutant), 200 pg of each of the two subunits of the GIRK channel (GIRK1 and GIRK2), and with 1 ng of the Gα_i3_ subunit. The oocyte was clamped to the desired holding potential in the ND96 solution (96 mM NaCl, 2 mM KCl, 1 mM CaCl_2_, 1 mM MgCl_2_, 5 mM Hepes, pH adjusted to 7.4 with NaOH). K^+^ currents were developed upon replacement of the ND96 by a 24 mM K^+^ solution (72 mM NaCl, 24 mM KCl, 1 mM CaCl_2_, 1 mM MgCl_2_, 5 mM Hepes, pH adjusted to 7.4 with KOH).

The expression of the M2R was evaluated, as described before [2], from measurements of [^3^H]Quinuclidinyl benzilate ([^3^H]QNB; specific activity 47.4 Ci/mmol, PerkinElmer, Waltham, MA) binding to intact oocytes. Expression level was ~18 fmol per oocyte in all experiments where either wt M2R or the mutant receptors were expressed.

For the PTX experiments, PTX protomer A (15 ng/oocyte; list biological laboratories, Campbell, CA) was injected to oocytes 12–20 hours before the experiment. The uncoupling of G-protein from the m2R following PTX treatment was verified before each experiment by two electrode voltage clamp measurements of acetylcholine (Sigma-Aldrich, Rehovot, Israel) induced GIRK currents [3].

### Statistical evaluation

Significance was checked by unpaired Student’s t test.

## Results and discussion

### The M2R exhibits constitutive activity

To investigate whether the M2R exhibits constitutive activity we used the muscarinic inverse agonist atropine. It was shown that constitutive activity in muscarinic receptors can be blocked by atropine [12,13]. Therefore, it is expected that if the M2R does exhibit constitutive activity, atropine will block it.

**Fig 1:**
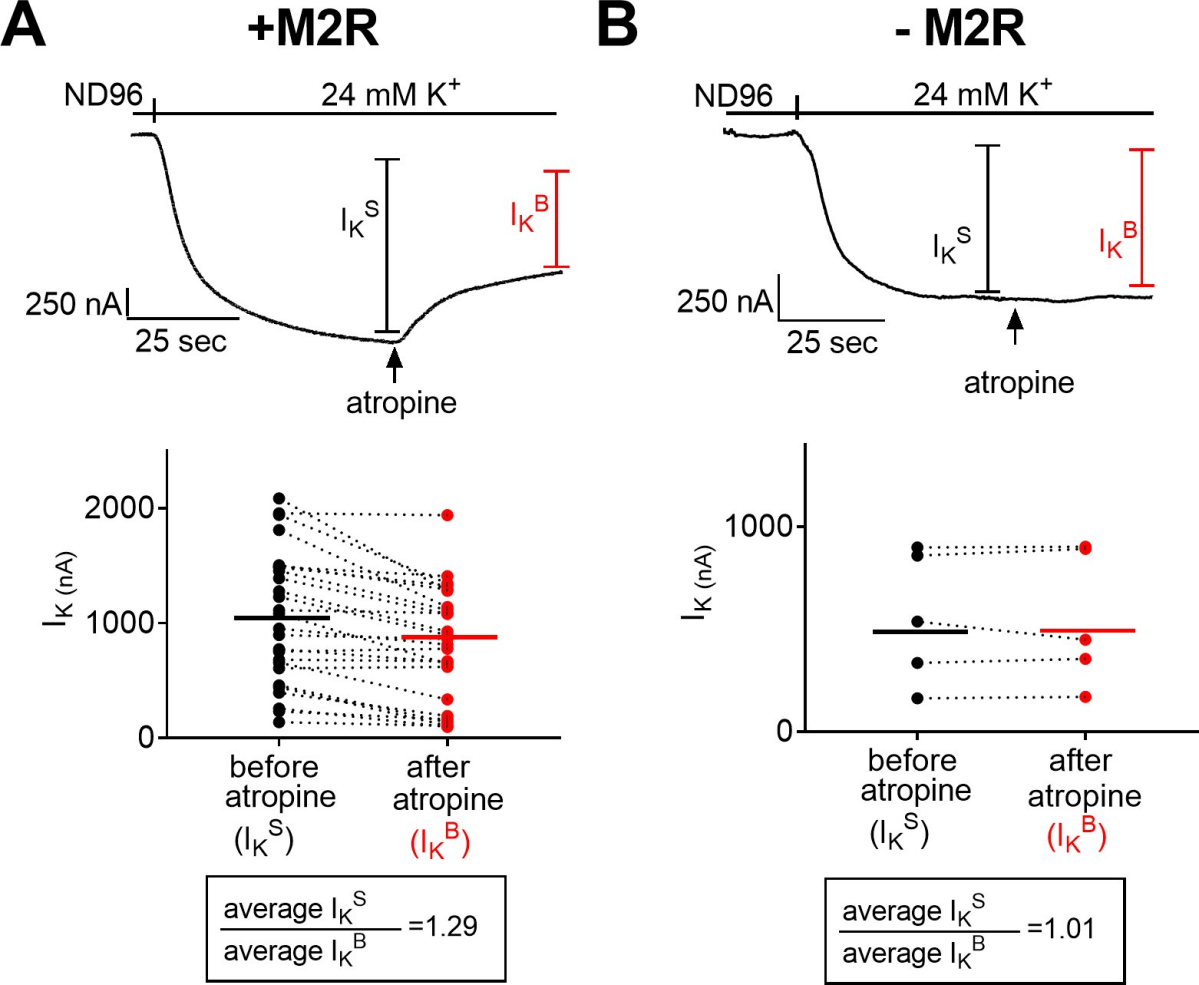
Measurement of constitutive activity in the M2R, using the atropine procedure. **(A)** The effect of 100 μM atropine on oocytes expressing both the M2R and GIRK channel. *(top)* A representative recording from one oocyte. *(bottom)* Collected data from 26 oocytes. Each two data points connected with a line represent I_K_^S^ (black) and I_K_^B^ (red), from one oocyte. The averages of I_K_^S^ and I_K_^B^ are denoted by the horizontal lines. The ratio I_K_^S^/ I_K_^B^ is given in the box. (**B**) The effect of 100 μM atropine on oocytes expressing only the GIRK channel. (*top*) A representative recording from one oocyte. (*bottom*) Data from 5 oocytes. Each two data points connected with a line represent I_K_ from one oocyte before (black) and after (red) the addition of atropine. The averages of I_K_ before and after the addition of atropine are denoted by the horizontal lines.

To examine whether this expectation is met we conducted the following experiment (Fig 1A, top). An oocyte expressing the M2R and the GIRK channel was voltage clamped to -80 mV in a low K^+^ solution (ND96, see Materials and Methods). Then, the solution was changed to high K^+^ (24 mM) and K^+^ currents (I_K_) were developed. The magnitude of I_K_ is calculated by subtracting the current amplitude in ND96, representing the background, GIRK-independent current, from the current amplitude in 24 mM K^+^.

Because the oocytes express both the M2R and the GIRK channel, the I_K_ that had developed is I_K_^S^, where I_K_^S^ = I_K_^B^ + I_K_^R^, where I_K_^B^ is the basal I_K_ produced by activation of the GIRK channel by the basal free Gβγ present in the oocyte [19] and I_K_^R^ is the additional I_K_ that is presumably produced by the constitutive activity of the M2R. Once I_K_^S^ reached a steady state, atropine (100 μM) was applied (Fig 1A, see arrow) and I_K_^S^ declined and reached a new steady state. Because 100 μM atropine completely block I_K_^R^ (see S1 Fig), this new steady state reflects I_K_^B^.

The experiment described above was repeated in 26 oocytes, and the results are depicted in Fig 1A, *bottom*. For each oocyte the amplitudes of I_K_^S^ (black circles) and I_K_^B^ (red circles) are shown, and the average of each is depicted by a horizontal line. In 24 of the 26 oocytes, the addition of atropine reduced I_K_^S^.

To check whether atropine affects the GIRK channel itself, we repeated the experiment of Fig 1A in oocytes expressing only the GIRK channel (Fig 1B). It is seen that in these oocytes the addition of atropine does not affect I_K_, indicating that atropine does not have an effect on the GIRK channel itself.

The results seen in Fig 1A, where I_K_^S^ declined following the addition of atropine in oocytes expressing the M2R, support the conclusion that the M2R exhibits constitutive activity (I_K_^R^) and that atropine indeed blocks it.

**Fig 2:**
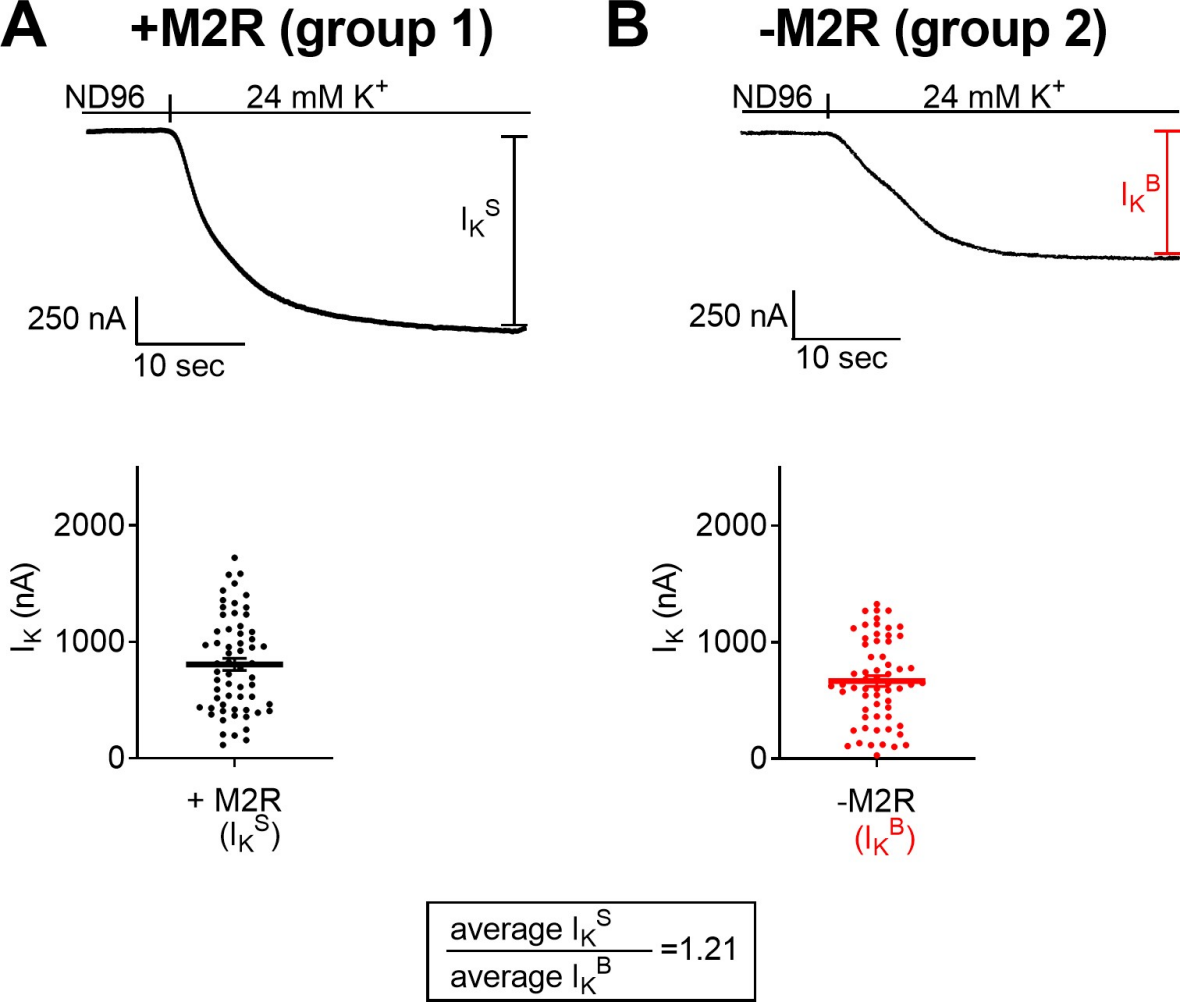
Measurement of constitutive activity in the M2R, using two groups of oocytes. **(A)** group 1: oocytes expressing both the M2R and the GIRK channel. (*top)* Representative recording. ***(****bottom****)*** Collected data from 61 oocytes. Here and in **B**, each data point depicts I_K_ measured from one oocyte, and the average I_K_ is denoted by the horizontal line. (**B**) group 2: oocytes expressing only the GIRK channel. (*top)* Representative recording. (*bottom*) Collected data from 61 oocytes. The ratio I_K_^S^/ I_K_^B^ is given in the box.

Another way, independent of the use of atropine, to examine whether the M2R exhibits constitutive activity is to compare I_K_ in two separate groups of oocytes: oocytes expressing both the GIRK channel and the M2R (group 1, Fig 2A) and oocytes expressing only the GIRK channel (group 2, Fig 2B). In group 1, I_K_ corresponds to I_K_^S^, i.e. the sum of I_K_^B^ and I_K_^R^, whereas in group 2, I_K_ corresponds only to I_K_^B^. We expect that if the M2R exhibits constitutive activity, then the amplitude of I_K_ in steady state will be higher in group 1 than in group 2.

To test whether the above expectation is met, oocytes from each group were voltage clamped to -80 mV, and I_K_ was measured, as described in Fig 1A. Fig 2A *top* and 2B *top* show examples of recordings from each group of oocytes. It is seen, that as expected, the amplitude of I_K_ in steady state is higher in group 1 than in group 2. Such recordings were repeated in 61 oocytes from each group and the cumulative results are seen in Fig 2A *bottom* and 2B *bottom*, where each circle represents one oocyte and the average is depicted by a horizontal line. It is seen that, as expected, the average I_K_ in group 1, which corresponds to I_K_^S^, is higher than the average I_K_ in group 2, which corresponds solely to I_K_^B^ (807±53 nA and 667±46 nA, respectively; the difference between the two groups is statistically significant, *p<0*.*05)*.

The results in Fig 2 further support the conclusion that the M2R exhibits constitutive activity.

In both types of experiments, the constitutive activity, I_K_^R^, could not be measured directly, but rather as a constituent of I_K_^S^. We are interested, however, in measuring I_K_^R^ quantitatively. This can be achieved by measuring the ratio I_K_^S^/ I_K_^B^ (see S1 Appendix). This ratio equals 1+ I_K_^R^/ I_K_^B^. Hence, it will be 1 when I_K_^R^ = 0 and it will be greater than 1 when constitutive activity takes place, in proportion to the extent of the constitutive activity.

In the experiment where atropine was used (Fig 1), the ratio between the average I_K_^S^ (Fig 1A *bottom*, black horizontal line) and the average I_K_^B^ (Fig 1A *bottom*, red horizontal line) was found to be 1.29 (Fig 1A, *box*). In the experiment shown in Fig 2, the average I_K_^S^ is extracted from oocytes of group 1 (Fig 2A) while the average I_K_^B^ is extracted from oocytes of group 2 (Fig 2B). This ratio was found to be 1.21 (Fig 2, *box*).

The resemblance in the values of the ratios obtained from the two types of experiments (1.29 in Fig 1A and 1.21 in Fig 2) implies that both methods are adequate for measuring and defining the extent of the constitutive activity.

### The constitutive activity of the M2R reflects its coupling to its G protein

**Fig 3:**
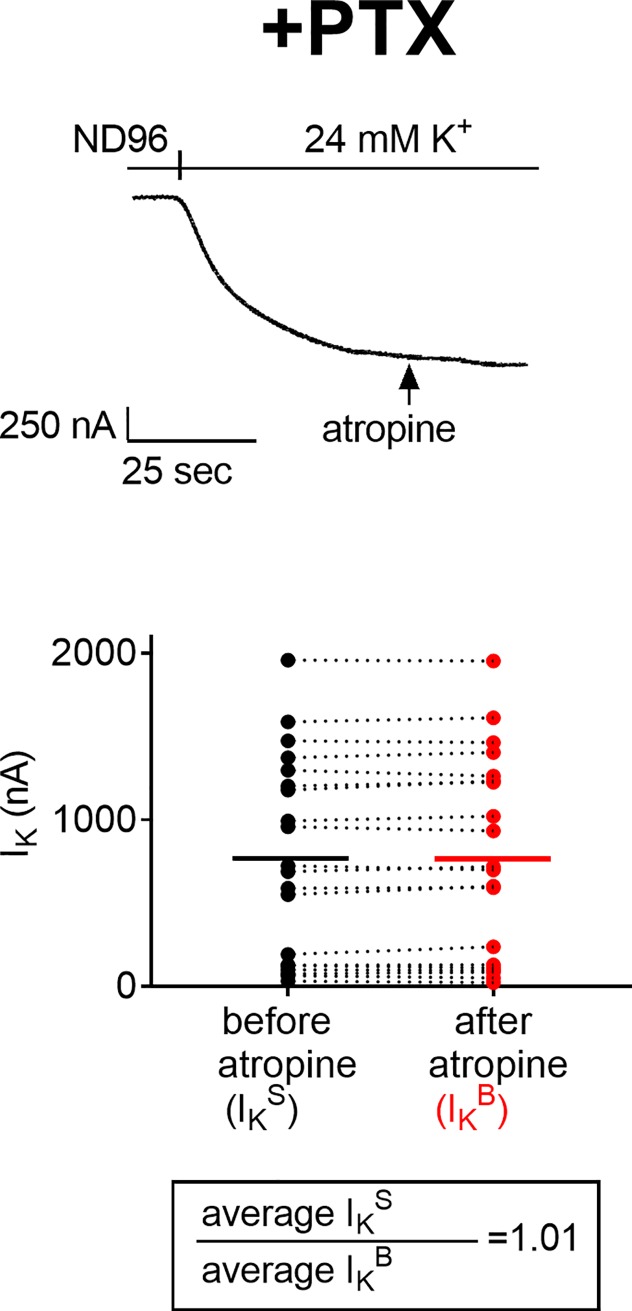
Constitutive activity in PTX treated oocytes. (*top*) Representative recording from a PTX-treated oocyte (solid line). Recording from control, PTX-untreated, oocyte is shown for comparison as dashed line. The arrows indicate the addition of atropine. *(bottom)* Collected data from 20 PTX-treated oocytes. Each two data points connected with a line represent I_K_ from one oocyte, before (black) and after (red) the addition of atropine. The averages of I_K_ before and after the addition of atropine are denoted by the horizontal solid lines. The averages of I_K_^S^ and I_K_^B^ from control, PTX-untreated, oocytes are shown for comparison as horizontal dashed lines. The ratio I_K_^S^/ I_K_^B^ from PTX-treated oocytes, is given in the box.

So far, we have shown (Figs 1 and 2) that the M2R exhibits constitutive activity. We now ask whether this constitutive activity (I_K_^R^) indeed reflects the coupling of the M2R to its G protein. To answer this question, we used pertussis toxin (PTX), which is known to uncouple G_o/i_ -coupled receptors, such as the M2R, from their G proteins. We expect that if the constitutive activity, I_K_^R^, reflects the coupling of the M2R to its G protein, then PTX will reduce it or even completely abolish it. To assess this expectation, we measured, using the atropine procedure, I_K_ in PTX-treated oocytes and compared it to I_K_ in oocytes not treated with PTX. We expect that in contrast to the effect of atropine in the control, PTX-untreated oocytes (Fig 1A, shown for comparison also as dashed line in Fig 3 *top*), atropine will have no effect in the PTX-treated oocytes. This is because in the PTX untreated oocytes I_K_ before the addition of atropine, I_K_^S^, is the sum of I_K_^B^+I_K_^R^. However, if PTX indeed abolishes I_K_^R^, then in the PTX-treated oocytes, I_K_ prior to the addition of atropine will reflect only I_K_^B^. Due to the same reason, I_K_ before the addition of atropine is expected to be higher in the PTX untreated oocytes than in the PTX treated ones.

Fig 3 shows that the expectations were met. Specifically, in contrast to PTX untreated oocytes (Fig 3 *top*, dashed line), in PTX-treated oocytes, atropine had no effect on I_K_ (Fig 3 *top*, solid line), compatible with the conclusion that in these oocytes constitutive activity did not take place. Furthermore, due to the same reason, I_K_ prior to the addition of atropine was lower in PTX-treated oocytes (Fig 3 *top*, solid line) than in PTX untreated oocytes (Fig 3 *top*, dashed line).

The experiment described above was repeated in 20 PTX-treated oocytes. It is seen (Fig 3 *bottom*), that in all 20 oocytes I_K_ after the addition of atropine (red circles) was the same as I_K_ prior to the addition of atropine (black circles), implying that atropine had no effect on I_K_. Corollary, the average I_K_ prior to the addition of atropine was lower in PTX-treated oocytes (764±134 pA, Fig 3 *bottom*, horizontal solid line) than in PTX untreated oocytes (1041±112 pA, Fig 3 *bottom*, horizontal dashed line).

To quantify the effect of PTX on the constitutive activity, we measured the ratio I_K_^S^/I_K_^B^ in PTX-treated oocytes. This ratio was found to be 1.01 (Fig 3, *box*), in comparison to the ratio of 1.29 in PTX-untreated oocytes (Fig 1A *box*).

The results in Fig 3 are compatible with the conclusion that the constitutive activity reflects the coupling of the M2R to its G protein (I_K_^R^). In the following, therefore, we will use "constitutive activity" and "coupling" interchangeably.

### The coupling between the M2R and its G protein is voltage dependent

We now wish to test our hypothesis that the coupling of the M2R to its G protein is voltage dependent. We recall, that the GIRK channel is by itself voltage dependent [19]. Thus, to tease apart the putative voltage dependence of the coupling from the voltage dependence of the GIRK channel, we compared I_K_ in two groups of oocytes. (1) Oocytes expressing the M2R and the GIRK channel. In these oocytes, I_K_ corresponds to the sum of I_K_^B^ and I_K_^R^ (I_K_^S^), and their voltage dependence reflects both the voltage dependence of the GIRK channel and the putative voltage dependence of I_K_^R^. (2) Oocytes expressing only the GIRK channel. In these oocytes, I_K_ corresponds only to I_K_^B^ and its voltage dependence reflects the voltage dependence of the GIRK channel *per se*.

**Fig 4:**
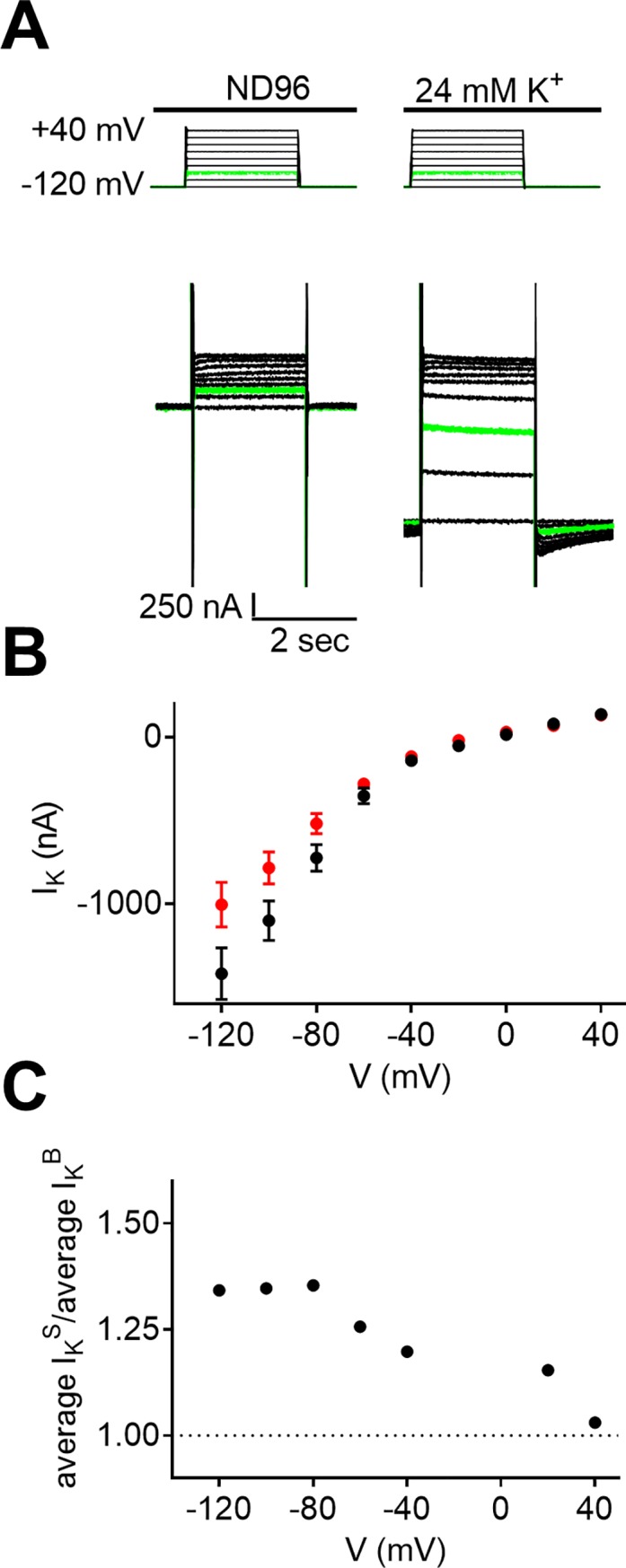
The voltage dependence of the constitutive activity of the M2R. **(A)** The experimental protocol (upper part) and the I_K_ produced at each holding potential (lower part). I_K_ was calculated by subtracting the current amplitude in ND96 from that in 24 mM K^+^ in the same holding potential. An example of recording in one holding potential (-80 mV) is shown in green. **(B)** I_K_^S^ (black) and I_K_^B^ (red) measured at various holding potentials. Each data point represents mean ±SEM from 69 oocytes. **(C)** The voltage dependence of the ratio I_K_^S^/I_K_^B^.

Thus, we measured I_K_ in the two groups of oocytes at various holding potentials ranging from -120 mV to +40 mV at 20 mV increments (Fig 4A). The magnitude of I_K_ at each holding potential was evaluated by subtracting the current amplitude in ND96 from that in 24 mM K^+^ at the same holding potential. The experiment was repeated in 69 oocytes from each group. Fig 4B shows I_K_^S^)black) and I_K_^B^ (red) at each holding potential. It is seen that at hyperpolarized potentials, the average I_K_^S^ (I_K_^B^+I_K_^R^) is higher than the average I_K_^B^ and this difference becomes smaller the higher the depolarization is.

The results in Fig 4B are compatible with the conclusion that the coupling (I_K_^R^) is voltage dependent.

In order to quantify I_K_^R^ and its putative voltage dependence, we measured the ratio between the average I_K_^S^ (from oocytes expressing both the M2R and the GIRK channel) and the average I_K_^B^ (from oocytes expressing only the GIRK channel) at each holding potential. We expect that if I_K_^R^ is voltage independent, then the ratio will also be voltage independent. On the other hand, if I_K_^R^ is voltage dependent then the ratio will also be voltage dependent; it will increase if I_K_^R^ increases at higher membrane potentials and decrease if I_K_^R^ declines at higher membrane potentials. The results are depicted in Fig 4C and show that the ratio I_K_^S^/I_K_^B^ declines at more positive membrane potentials, supporting the conclusion that the coupling of the M2R to its G protein is voltage dependent. Moreover, the coupling is stronger at hyperpolarization than under depolarization.

### The same voltage sensor controls the voltage dependence of both the agonist binding and the coupling

We next ask what controls the voltage dependence of the coupling of the M2R to its G protein.

We had shown that the voltage dependence of binding of acetylcholine (ACh) to the M2R is controlled by a voltage sensor, composed of three tyrosine residues (Tyr104, Tyr403, Tyr426), that is situated in the agonist binding site [18]. We expect that if this voltage sensor controls also the coupling, then the agonist binding and the coupling will show similar voltage dependence. Furthermore, disrupting the voltage sensor will affect similarly both the agonist binding and the coupling.

**Fig 5:**
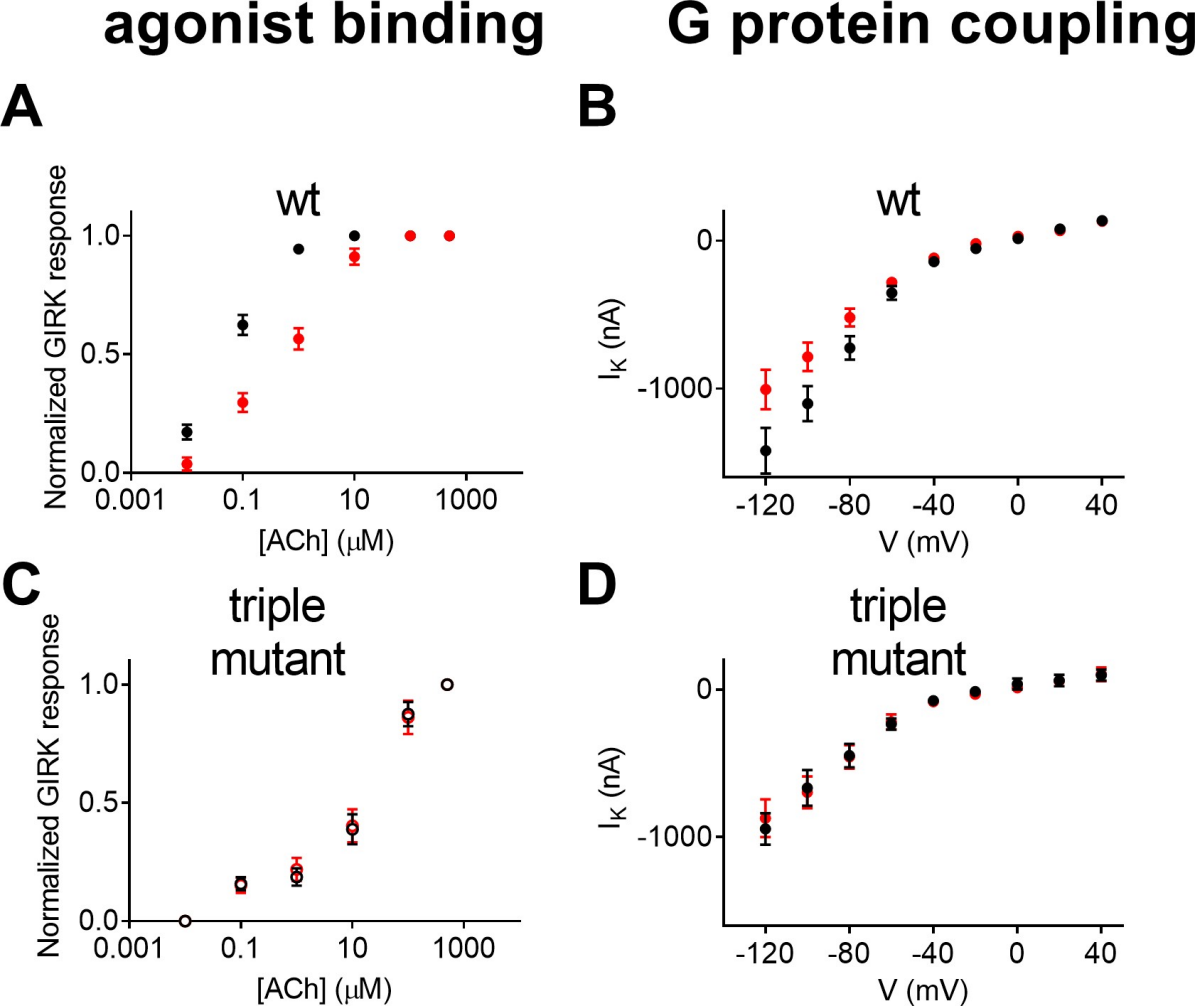
Voltage dependence of the binding affinity and the constitutive activity of the wt M2R and the triple mutant. **(A**) Dose-response curves obtained from several experiments at -80 mV (black circles) and at +40 mV (red circles) using various concentrations of ACh from wt M2R. Each data point represents the mean ± SEM, n = 5–8; taken from [18]. **(B)** I_K_^S^ (black) and I_K_^B^ (red) measured at various holding potentials from wt M2R; taken from Fig 4B. (**C)** Dose-response curves from the triple mutant at -80 mV (black circles) and at +40 mV (red circles). Each data point represents the mean ± SEM, n = 6–8. **(D)** I_K_^S^ (black circles) and I_K_^B^ (red circles) measured at various holding potentials. Each data point represents mean ±SEM from 21 oocytes.

Fig 5 shows that this is indeed the case. As seen, in wt M2R both the agonist binding (Fig 5A, taken from [18]) and the coupling (Fig 4B, shown for comparison also in Fig 5B) decline at more positive membrane potentials.

We next examined whether disrupting the voltage sensor will have similar effects on the voltage dependence of both the agonist binding and the coupling. To do so we compared the voltage dependence of the agonist binding to the voltage dependence of the coupling in a mutant where the three tyrosine residues of the voltage sensor were replaced by alanine, denoted the triple mutant [18]. Binding of ACh to the triple mutant was measured as described before [3,18]. Specifically, dose-response curves were measured at two holding potentials, -80 mV and +40 mV, using ACh-induced GIRK currents as a measure for ACh binding. As before, to enable comparison between different holding potentials, the response to each concentration of ACh was normalized to the maximal response evoked by ACh at the same holding potential. Fig 5C shows that in contrast to the case of wt M2R, where the binding affinity was voltage dependent [3], in the triple mutant the voltage dependence was abolished; the binding affinity at hyperpolarization was as low as under depolarization. To examine the effect of the triple mutant on the voltage dependence of the coupling we repeated the experiment described in Fig 4A using oocytes expressing the triple mutant. Fig 5D shows that in contrast to the case in the wt, in the triple mutant the voltage dependence of the coupling disappeared; the coupling at hyperpolarization was as low as under depolarization.

The results seen in Fig 5 support the conclusion that the same voltage sensor controls the voltage dependence of both the agonist binding and the coupling.

We showed here, for the first time, that the coupling of a prototypical GPCR, the M2R, to its cognate G protein is voltage dependent. This conclusion is based on the observation that the constitutive activity of the M2R, which we showed to reflect the coupling, is voltage dependent.

GPCRs mediate most signal transduction processes. Specifically, the binding of an external agonist promotes coupling of the GPCR to its cognate G protein and this, in turn, induces downstream signaling.

In recent years, it was shown that GPCRs are voltage sensitive; for several GPCRs it was shown that their binding affinity is voltage dependent [3,7,11,20].

Here, we make a step forward and show that in addition to the binding of agonist, also the actual execution of signal transduction, which is initiated by coupling of the GPCR to its G protein, is voltage dependent. This finding is of utmost physiological importance as it implies that voltage actually controls signal transduction.

## Supporting information

S1 Appendix (DOCX)

S1 Fig Determining the atropine concentration that produces maximal block of the constitutive activity. (DOCX)
