# Supplementary material for: The coupling of the M2 muscarinic receptor to its G protein is voltage dependent
Source: PLoS One. 2019 Oct 31;14(10):e0224367. doi: 10.1371/journal.pone.0224367 (PMC6822938; doi:10.1371/journal.pone.0224367)
Supplement: S1 Appendix — (DOCX) [file pone.0224367.s001.docx]

**Supporting Information**

**The Coupling of the M2 muscarinic receptor to its G protein is voltage dependent**

Yair Ben-Chaim, Chava Broide, and Hanna Parnas

**Appendix**

**The ratio I_K_^S^/I_K_^B^**

We measure the putative constitutive activity of the M2R by the free Gβγ subunits that are produced upon the spontaneous activation of the M2R in the absence of agonist. Activation of the M2R results in dissociation of the coupled G protein to Gα and Gβγ Subunits. The free Gβγ subunits open the GIRK channel, leading to the evolvement of K^+^ currents (I_K_).

In oocytes expressing only the GIRK channel I_K_ will be produced by the basal level of Gβγ, hence this I_K_ is denoted I_K_^B^. In oocytes expressing both the GIRK channel and the M2R, I_K_ will be the sum (hence denoted I_K_^S^) of I_K_^B^ and the additional I_K_ produced by the receptor, denoted I_K_^R^. Hence, I_K_^S^=I_K_^B^+I_K_^R^.

Here we ask whether I_K_^R^ is voltage dependent. In an attempt to answer this question, we encounter two problems: 1. the GIRK channel itself is voltage dependent. i.e. its current-voltage relationship (i-v) is linear at negative membrane potentials and it levels off at more positive potentials. Therefore, one needs to develop tools to separate between the voltage dependence of the GIRK channel and the putative voltage dependence of I_K_^R^.

2. I_K_^R^ cannot be measured directly. This is because in oocytes expressing both the M2R and the GIRK channel the evolved current, I_K_^S^, is the sum of I_K_^B^ and I_K_^R^.

We suggest overcoming these two problems by measuring the ratio between I_K_^S^ and I_K_^B^. This ratio is given by:

(1)$\frac{I_{K}^{S}}{I_{K}^{B}}$=$\frac{I_{K+}^{B}I_{K}^{R}}{I_{K}^{B}}$=1+$\frac{I_{K}^{R}}{I_{K}^{B}}$

If I_K_^S^ and I_K_^B^ are measured at the same membrane potential, the ratio between I_K_^S^ and I_K_^B^ directly reflects I_K_^R^. Specifically, if I_K_^R^=0, the ratio will be 1 and it will be higher than 1 as I_K_^R^ increases.

The question is whether the ratio still reflects I_K_^R^ if the experiments are done at varying membrane potentials. Eq. 1 shows that the answer is positive providing that I_K_^B^ and I_K_^S^ are measured at the same membrane potential.

In conclusion, to answer the question whether I_K_^R^ is voltage dependent, one should measure, in the same experiment, I_K_^S^ and I_K_^B^ at various holding potentials where I_K_^S^ is extracted from oocytes expressing both the GIRK channel and the M2R and I_K_^B^ is extracted from oocytes expressing only the GIRK channel.
