# Supplementary material for: The coupling of the M2 muscarinic receptor to its G protein is voltage dependent
Source: PLoS One. 2019 Oct 31;14(10):e0224367. doi: 10.1371/journal.pone.0224367 (PMC6822938; doi:10.1371/journal.pone.0224367)
Supplement: S1 Fig — (DOCX) [file pone.0224367.s002.docx]

**Supporting Information**

**The Coupling of the M2 muscarinic receptor to its G protein is voltage dependent**

Yair Ben-Chaim, Chava Broide, and Hanna Parnas

**Figure S1.** Determining the atropine concentration that produces maximal block of the constitutive activity. In order to determine the concentration of atropine that will be used in the experiments shown in Fig 1 and Fig 3 the effect of different atropine concentrations (10, 100 and 200 µM) on I_K_^S^ was measured as described in Fig 1A. It is seen that increasing atropine concentration from 100 µM to 200 µM did not have further effect on I_K_^S^, suggesting that 100 µM was sufficient in order to produce maximal block of the constitutive activity. Following this experiment, 100 µM atropine was used in the experiments described in Fig 1 and in Fig 3.
